# Effect of Antiresorptive Drugs on Osseointegrated Dental Implants: A Systematic Review

**DOI:** 10.3390/jcm13072091

**Published:** 2024-04-03

**Authors:** Joyce Tin Wing Li, Yiu Yan Leung

**Affiliations:** Oral and Maxillofacial Surgery, Faculty of Dentistry, The University of Hong Kong, Pok Fu Lam, Hong Kong; litinwingjoyce@gmail.com

**Keywords:** dental implant, osseointegration, antiresorptive drug, bisphosphonate, denosumab, medication-related osteonecrosis of the jaw, systematic review

## Abstract

**Background:** This systematic review aimed to evaluate the impact of antiresorptive drug therapy on osseointegrated dental implants and the association with medication-related osteonecrosis of the jaw (MRONJ). **Methods:** A systematic search, including a computer search of several databases with specific keywords, a reference search, and a manual search of four key maxillofacial journals were performed. Relevant articles were then evaluated and those that fulfilled the five predetermined criteria were chosen to enter the final review. A total of 445 implants in 135 subjects were included in the eight studies analyzed in the final review. **Results:** The failure rate of dental implants after antiresorptive medication in the included studies was 23%, with 83% of failures attributed to MRONJ. The average time from antiresorptive drug initiation to MRONJ development was approximately 34 months, ranging from 3 months to 16 years. The majority of MRONJ cases were classified as stage 2, and all sites showed either complete healing or substantial mucosal coverage after treatment. **Conclusions:** This review highlights the significant impact of antiresorptive drugs on osseo- integrated implants, with MRONJ identified as a leading cause of implant failure. The potential role of peri-implantitis as a trigger for MRONJ is emphasized. Regular monitoring and maintaining good periodontal health, especially within the first three years of antiresorptive drug therapy initiation, are crucial for implant success. Physicians and dentists should provide comprehensive information to patients prescribed with antiresorptive drugs, emphasizing the need for an awareness of the risks of MRONJ in the context of osseointegrated implants. A longer term of follow-up is recommended to identify and manage MRONJ around dental implants in an early manner.

## 1. Introduction

Antiresorptive drugs are commonly prescribed to reduce the risk of fractures associated with osteoporosis, as well as in patients with skeletal involvement from advanced malignancies such as breast, lung, prostate cancer, and multiple myeloma [1]. However, the use of these medications has been associated with a potential complication known as medication-related osteonecrosis of the jaw (MRONJ).

Both animal and human studies have indicated that the combination of antiresorptive medication and inflammation or infection is necessary and sufficient to induce MRONJ [2]. MRONJ is a severe and long-lasting condition characterized by exposed necrotic bones in the jaw. Bisphosphonates and denosumab, which are antiresorptive medications, directly affect osteoclasts, leading to decreased bone remodeling and inhibition of bone resorption. The annual incidence of MRONJ has been reported to be higher in individuals receiving high-dose antiresorptive therapy (2305.8 cases per 100,000 individuals) compared to those receiving low-dose therapy (132.5 cases per 100,000 individuals) [3]. In comparison, the incidence of osteonecrosis of the jaw among individuals not taking antiresorptive agents is significantly lower at 5.1 cases per 100,000 individuals [3]. Several studies have also demonstrated an association between MRONJ and the use of bisphosphonate treatment [4,5,6].

The treatment of MRONJ can be challenging and time-consuming, highlighting the importance of determining the optimal timing for dental implant placement in patients who plan to receive antiresorptive drugs or have a family history of osteoporosis. This is crucial to optimize implant outcomes and prevent potential complications. One of the main challenges in providing dental care to patients on antiresorptive drugs is the lack of awareness among physicians regarding the effects of these medications on jaw osteonecrosis [7,8]. Dental implants require proper osseointegration, but antiresorptive drugs can inhibit bone turnover and potentially interfere with the osseointegration process.

As the field of implantology has gained popularity, numerous studies have explored the relationship between various factors, such as diabetes [9], periodontitis [10], and osteoporosis [11], and their impact on implant survival rates. However, there is a noticeable dearth of research specifically investigating the effects of antiresorptive drugs on osseointegrated implants. This systematic review aimed to evaluate the effects of antiresorptive drugs on osseointegrated implants and provide evidence-based guidelines for dental practitioners. This included examining the characteristics of MRONJ implants and sites, as well as considering factors such as the type of drug, dosage, duration of therapy, and the patient’s risk profile. By addressing these aspects, this review aimed to contribute to the understanding of dental implant outcomes in patients who plan to receive antiresorptive therapy and to guide clinical decision-making.

## 2. Materials and Methods

### 2.1. Protocol

A systematic review was performed following the preferred reporting items for systematic reviews and meta-analyses (PRISMA 2020) guidelines [12]. The systematic review was not registered in PROSPERO.

### 2.2. Focused Questions

The review was structured using the PICO design as in Table 1.

### 2.3. Search Strategy

A comprehensive search was conducted in two electronic databases, PubMed and Embase, covering the period from their inception to 16 October 2023. The key terms for the searches were “dental implant” AND “antiresorptive” OR “bisphosphonate” OR “denosumab” OR “osteonecrosis” OR “medication-related”. There were no restrictions on article language. The last date of the search was 16 October 2023. Additionally, several international journals such as the *International Journal of Oral and Maxillofacial Surgery* (*IJOMS*), the *Journal of Oral and Maxillofacial Surgery* (*JOMS*), the *Journal of Craniofacial Surgery*, and the *International Journal of Oral & Maxillofacial Implants* (*JOMI*) were manually searched for additional studies. The manual search of these journals was limited to the period from 2011 to 2023. Articles relevant to dental implants and antiresorptive drugs were selected. The reference lists of relevant articles were also reviewed.

### 2.4. Study Selection

Two independent reviewers, both experienced in data collection and data analysis, underwent training on the process, which included database searches, the proper identification of study design and eligibility criteria, and accurate data extraction and entry. Both screened the titles and abstracts of the retrieved articles to determine eligibility. The inclusion criteria were as follows: (1) the study was a clinical trial or a case series involving at least 5 dental implants in human subjects who were taking antiresorptive drugs, and provided clear documentation of the reason for drug use, as well as detailed information on the type and route of drug administration; (2) the implant was inserted and osseointegrated before the initiation of antiresorptive drug therapy; (3) the average time from the initiation of antiresorptive drug therapy to the diagnosis of MRONJ was recorded; (4) MRONJ treatment was mentioned; and (5) there was a follow-up period of at least six months post MRONJ diagnosis (or four years if not diagnosed). Studies that did not meet the inclusion criteria were excluded. In the second step, all full articles were evaluated. Discrepancies were resolved through discussion, and a third party was consulted if consensus could not be reached.

### 2.5. Data Extraction

Data were extracted from the included studies using a standardized data extraction form. The extracted data included study characteristics (e.g., authors, year of publication, and study design), participant characteristics (e.g., sample size, age, gender, and demographics), intervention details, outcome measures used, and relevant results. Data extraction was performed independently by two reviewers, and any discrepancies were resolved through discussion.

### 2.6. Quality Assessment

The methodology quality and risk of bias of the included studies were assessed. Two reviewers independently evaluated the quality of each study, considering factors such as the presence of a control group, blinding, sample size, a representative sample, standardized data collection, complete data reporting, objective outcome measures, consistent outcomes, appropriate statistical tests, and the complete reporting of results. Any disagreements were resolved through discussion.

### 2.7. Data Synthesis and Analysis

Due to anticipated heterogeneity among the included studies, a meta-analysis was not feasible. Therefore, a narrative synthesis of the findings was conducted. The results were thematically organized and descriptively summarized. Subgroup analyses based on participant characteristics were planned and conducted where appropriate.

## 3. Results

### 3.1. Study Selection and Characteristics

**Figure 1:**
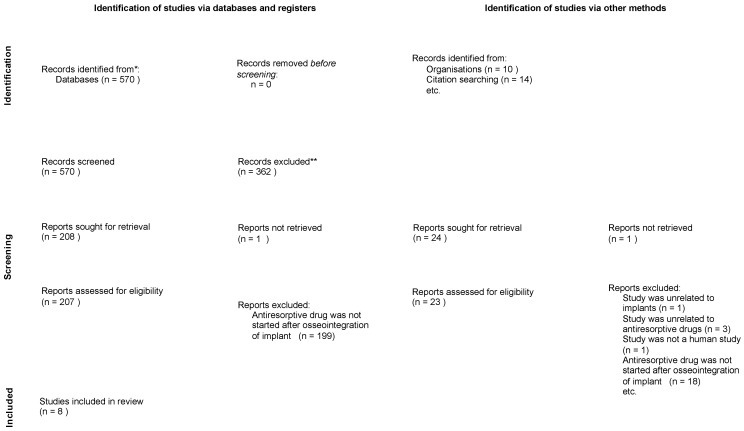
Flow diagram for article selection (as adopted from the PRISMA 2020 flow diagram [12]). * Databases searched were PubMed and Embase; ** Record excluded based on irrelevance to the topic of antiresorptive drug to the dental implants.

The flow diagram illustrating the three rounds of searches and evaluation is presented in Figure 1. Using the predetermined key terms, a total of 565 hits were generated from PubMed, with an additional 5 hits from Embase. The abstracts of these articles were screened, and 208 articles were deemed relevant to the study on the effectiveness of antiresorptive drugs on dental implants, thus advancing to the next round of evaluation. Of these, 1 report could not be retrieved, and in 199 reports, antiresorptive drugs did not commence after the osseointegration of implants.

A manual search of the 10 most recent years of four international journals yielded 10 additional articles. Furthermore, a search of the reference lists of the included articles from the first round resulted in the identification of 14 more articles that were potentially relevant to the effectiveness of antiresorptive drugs on osseointegrated implants. Of these, 1 could not be retrieved, and the full texts of the remaining 23 articles were obtained. Among the 23 articles, 1 was excluded as it did not pertain to implants and 1 was not a human study, while 3 and 18 studies were excluded for being unrelated to antiresorptive drugs and for not initiating antiresorptive drug treatment after the osseointegration of the implant, respectively.

Out of the 230 articles, 222 studies failed to meet at least one of the criteria and were subsequently excluded. Ultimately, eight studies fulfilled all the criteria and progressed to the final round of evaluation.

The articles included in the final review are presented in Table 2. Among them, seven studies were case series, while only one study was a retrospective cohort study. No randomized controlled clinical trials were identified. Additionally, all included studies were published in English.

### 3.2. Risk of Bias in Studies

The risk of bias assessment in individual studies is summarized in Table 3. Most of the included studies were case series (seven out of eight) with a moderate risk of bias.

### 3.3. Result of Syntheses

#### 3.3.1. Demographics (Table 2)

A total of 445 implants in 135 subjects were included in the eight studies analyzed. Gender distribution data were reported in six studies, which included 37 female patients and 4 male patients. The reported median age was 65 years (ranging from 42 to 83 years). In total, 445 dental implants were included in this systematic review. Out of these, 77% (344/445) were successful, while the remaining 23% (101/445) failed. Out of the 92 implant failures analyzed (9 failed implants were not reported for the cause of failure), 83% of the implant (76/92) failures were attributed to medication-related osteonecrosis of the jaw (MRONJ), while the remaining 17% (16/92) were due to other reasons, primarily peri-implantitis. All MRONJ studies had a minimum follow-up period of at least six months postoperatively.

#### 3.3.2. Average Time from Initiation of Antiresorptive Drug to MRONJ (Table 2)

The time interval between the initiation of antiresorptive drug therapy and the diagnosis of MRONJ varied widely, ranging from 3 months to 192 months (equivalent to 16 years). All eight articles reported a mean duration of 34 months between the initiation of antiresorptive drug therapy and the diagnosis of MRONJ.

#### 3.3.3. Antiresorptive Drug (Table 4)

Four studies (Goss [15], Massaad [16], Kwon [17], and Shabestari [19]) provided information on the reasons for administering antiresorptive drugs. Out of a total of 27 subjects, 25 subjects were prescribed antiresorptive drugs due to osteoporosis, while the remaining 2 subjects were receiving treatment for cancer-related conditions. Regarding the specific types of antiresorptive drugs used, 66 subjects were prescribed bisphosphonates, while 9 subjects were administered denosumab. Among the bisphosphonate users, 33 subjects received alendronate, 18 subjects were prescribed zoledronate, and 1 subject received risedronate, whereas the specific drug was not mentioned for the remaining subjects. In terms of administration route, orally administered antiresorptive drugs were more common, with 54 out of 89 subjects receiving them in this manner. Intravenous or subcutaneous administration was noted in 35 cases (39%) out of the total subjects.

**Table 4:** Summary of demographics of participants and their outcomes.

Study	Patients	Mean Age (Years)	Gender	Diagnosis	Type of ARD	Route of Administration	Follow-Up Period	Number of Implants	Survival	Failure	Failure Reason
Pichardo 2020 [13]	1–14	NS	NS	NS	NS	6 O 5 IV 3 S	≥3 months postoperatively after MRONJ treatment; median 12.5 (3–36) months	34	8	26	26 MRONJ
Holzinger 2014 [14]	15–17	NS	3 F	NS	All BPs 3 Z/I	3 IV	Retrospective study, studies from 2004 to 2012	8	0	8	8 MRONJ
Goss 2010 [15]	18–21	70 (66–75)	2 M, 2 F	All OS	All BPs 3 A 1 A then R	4 O	Up to 3 years post surgery	12	6	6	4 MRONJ 2 Fell out
Massaad 2022 [16]	22–27	66 (50–83)	2 M, 4 F	4 OS 2 C	3 D 2 Z 1 A/Z	1 O, IV 2 IV 3 S	Minimum twice/month until improvement of symptoms/healing; 4 stable, 7 healed	11	0	11	11 MRONJ
Kwon 2014 [17]	28–30	70 (67–73)	3 F	All OS	All BPs 2 A 1 R	3 O	Until lesion completely/mostly covered by mucosa	5	0	5	5 MRONJ
Pogrel 2018 [18]	31–41	NS	11 F	NS	D/BP 8 A 1 Z 2 D	8 O 1 IV 2 S	≥2 years	11	0	11	11 MRONJ
Shabestari 2010 [19]	42–55	53 (42–79)	14 F	All OS	All BPs	14 O	Up to 4 years	20	20	0	NA
Kim 2020 [20]	56–135	67.7	30 M, 314 F (in terms of implant number)	NS	12 Z 18 A/R 4 D/I	18 O 12 IV 4 S	≥1 year	344	310	34	11 MRONJ 14 Peri-implantitis
Total	135	65 (42–83)	9% M 34/385 91% F 351/385	93% OS 25/27 7% C 2/27	88% BPs (66/75) 12% D (9/75)	61% O (54/89) 39% IV/S 35/89	≥1 year	445	77% 344/445	23% 101/445	83% MRONJ 76/92 17% Peri-implantitis 16/92

NA: Not Applicable; NS: Not Specified; Gender: M—Male; F—Female; Diagnosis: C—Cancer; OS—Osteoporosis; Type of ARD: A—Alendronate; BP—Bisphosphonate; D—Denosumab; I—Ibandronate; R—Risedronate; Z—Zoledronate; Route of Administration: O—Orally; IV—Intravenously; S—Subcutaneously.

#### 3.3.4. Location of MRONJ (Table 5)

Among the 31 failed dental implants attributed to MRONJ, 36% (11/31) occurred in the maxilla, while the remaining 65% (20/31) were located at the mandible. In sum, 85% (17/20) occurred in the posterior region, and 15% (3/20) occurred in the anterior region.

**Table 5:** Summary of failed dental implants due to MRONJ.

Study	Implants w/MRONJ	ARD-MRONJ Mean Time (Months)	Reasons	Level of MRONJ	Site of MRONJ	Site of Survived Implants
Pichardo 2020 [13]	1–26	median 24 (7–120)	26 MRONJ	NS	NS	
Holzinger 2014 [14]	27–34	18 (14–23)	8 MRONJ	NS	NS	
Goss 2010 [15]	35–38	34 (3–61)	4 MRONJ	2—Extensive ONJ 2—Localized ONJ	3 posterior mand 1 anterior mand	6 max
Massaad 2022 [16]	39–49	51 (24–192)	11 MRONJ	10—Stage 2 1—Stage 3	3 posterior max 2 anterior max 6 posterior mand	
Kwon 2014 [17]	50–54	19 (13–27)	5 MRONJ	NS	4 posterior max 1 posterior mand	
Pogrel 2018 [18]	55–65	58 (24–156)	11 MRONJ	NS	2 max 9 mand	
Shabestari 2010 [19]	NA	NA	NA	NA	not applicable	3 posterior max 5 anterior max 5 posterior mand 7 anterior mand
Kim 2020 [20]	66–76	11—≤12 months 10—13–35 months 13—≥36 months	11 MRONJ	NS	34 MRONJ/Peri-implantitis 44% max (15/34) 56% mand (19/34) 3% anterior (1/34) 38% premolar (13/34) 59% molar (20/34)	
Total	76	range 3–192	MRONJ (76/92)			

NA: not applicable; NS: not specified; max: maxilla; mand: mandible.

#### 3.3.5. Severity of MRONJ (Table 5)

Two studies (Goss [15], and Massaad [16]) provided information on the severity of MRONJ, involving a total of 15 dental implants. Overall, 12 failed implants were classified as stage 2 MRONJ, while 3 implants were classified as stage 3 MRONJ. However, the remaining studies did not specify the level of MRONJ for the failed implants.

#### 3.3.6. Treatment Modalities of MRONJ (Table 6)

Treatment approaches were consistent across all studies and included surgical procedures such as sequestrectomy, curettage, and explantation. Additionally, systematic antibiotics such as amoxicillin and clavulanic acid were commonly prescribed. In summary, 27% of the dental implants could be preserved, 75% of the MRONJ sites were completely healed, and the remaining sites were stable and mostly covered by mucosa. No unhealed sites or serious complications were mentioned.

**Table 6:** Treatment modalities.

Study	Implants w/MRONJ	Treatments	Outcome of Treatment
Pichardo 2020 [13]	1–26	Mostly treated with sequestrectomy and antibiotics	A total of 94% had closed and healed mucosa and were free of complaints. A total of 36% of the implants could be preserved.
Holzinger 2014 [14]	27–34	Implant removed during ostectomy for osteonecrosis	A total of 36% of the implants could be preserved.
Goss 2010 [15]	35–38	2 attempted surgeries with antibiotics 1 surgical salvation 1 removed 1 nil	A total of 75% healed over 3 months; only one case had pain for 3 years, which was then resolved.
Massaad 2022 [16]	39–49	Local (CHX, H_2_O_2_ mouthwash) Antibiotics (Amoxicillin, Clavulanic acid) Surgical procedures (explantation, curettage, and sequestrectomy)	A total of 17% of the implants could be preserved. A total of 64% of the MRONJ locations were healed, and 46% were stable.
Kwon 2014 [17]	50–54	Sequestrectomy, removed implant, curettage	A total of 20% of the implants could be preserved. A total of 40% of the MRONJ sites were completely covered by mucosa, and 60% were mostly covered by mucosa.
Pogrel 2018 [18]	55–65	Antibiotics to all, 8 implants removed, curettage	All cases had satisfactory healing following implant removal and debridement.
Shabestari 2010 [19]	NA	No treatment needed	No treatment needed.
Kim 2020 [20]	66–76	Removed simultaneously during removal of sequestrum in MRONJ	Not specified.
Total			A total of 27% of the implants could be preserved. A total of 75% of the MRONJ sites were completely healed.

## 4. Discussion

The key findings of this systematic review were that (1) 23% of the dental implants in the included studies failed after antiresorptive treatment. (2) Among the failed implants, the majority (83%) of the failures were attributed to MRONJ. (3) The average time between the initiation of antiresorptive drugs and the development of MRONJ was found to be 34 months. (4) A total of 65% of the implants that failed due to MRONJ were located in the mandible, with 85% occurring in the posterior region. (5) The majority of the MRONJ cases (80%) were classified as stage 2, indicating that the necrotic bone was confined to the alveolar bone region.

This systematic review aimed to provide an objective summary of the current knowledge regarding the outcomes of osseointegrated implants following the initiation of antiresorptive drug therapy, as well as the treatment modalities for MRONJ. However, we found a scarcity of systematic reviews on the subject, with most of the available literature consisting of case series.

As the popularity of dental implant procedures continues to rise, different dental associations have provided guidelines to assist dentists in managing patients who use antiresorptive drugs. The European Federation of Periodontology (EFP) [21] advises against implant placement in individuals taking bisphosphonates or denosumab due to the heightened risk of MRONJ. However, the American Dental Association (ADA) [2] suggests that nonmalignant disease patients undergoing antiresorptive therapy may proceed with their operative plan, with a careful consideration of factors such as drug schedule and the duration of therapy. In contrast, malignant disease patients are contraindicated for dental implants during antiresorptive therapy. The National Health Service (NHS) [22] suggests that patients receiving intravenous bisphosphonates are generally not suitable candidates for implant treatment due to the higher known risk of bone necrosis. On the other hand, patients on short-term oral bisphosphates have a lower risk of bone necrosis. One area that remains unclear is the chance of MRONJ and the management of patients who initiate antiresorptive drug therapy after implant placement and full osseointegration.

This review aligns with studies by Jacobsen [23] and Lazarovici [24], which found a higher risk of osteonecrosis of the jaws associated with posteriorly placed dental implants. Lazarovici [24] also highlighted that two-thirds of MRONJ cases occurred in the mandible, confirming the risks associated with dental implants in the posterior mandible. Therefore, careful consideration should be given to placing dental implants in this area. Regular recall visits and close monitoring are crucial for promptly identifying any complications or adverse events.

Regarding the dental implant failure rate, this systematic review revealed a rate of 23% in osseointegrated implants following the initiation of antiresorptive drugs. The average duration between the initiation of antiresorptive drug therapy and the diagnosis of MRONJ was found to be 34 months, emphasizing the critical importance of close monitoring during the first three years following the initiation of antiresorptive drug therapy.

In this study, it was observed that the majority of MRONJ cases were caused by oral antiresorptive therapy rather than intravenous bisphosphates (IV BPs). This finding challenges the notion of an absolute contraindication to dental implant treatment for IV BP users. Previous studies, such as those by Scully et al. [25] and Serra et al. [26], have proposed this contraindication due to the increased risk of MRONJ, but there are divergent opinions on this topic. The findings of this systematic review suggest that a blanket ban may not be necessary, and the decision to proceed with dental implants in IV BP users should be made on a case-by-case basis, considering individual risk profiles, overall health status, and potential treatment benefits. Close collaboration between dental and medical professionals is crucial to assess the risks, benefits, and alternatives for each patient.

A study (Seki et al. [27]) has suggested that peri-implantitis may serve as a trigger for MRONJ [28], even in cases where the implant is already osseointegrated. The presence of peri-implantitis, characterized by inflammation and bone loss around the implant, can create a favorable environment for the development of MRONJ. Research has revealed significant associations between clinical/radiographic signs of peri-implantitis and the occurrence of peri-implant MRONJ [29]. The diagnosis of peri-implantitis was neither related to the underlying disease (osteoporosis/oncology) nor to the route of antiresorptive administration and might therefore be an independent risk factor for MRONJ development [29]. Additionally, a study (Kim et al. [20]) has identified pre-existing marginal bone loss around dental implants as a risk factor for implant survival. This finding suggests that patients with pre-existing marginal bone loss may be at a higher risk of implant failure when undergoing antiresorptive drug therapy. This highlights the importance of comprehensive implant evaluation and management before initiating antiresorptive therapy.

It is worth noting that MRONJ predominantly affects the area around the dental implant rather than the adjacent tissues, as observed in the study by Pogrel et al. [18]. The localized nature of MRONJ around the implant can make it more manageable when it occurs. Recognizing this impact allows dental practitioners to focus interventions and treatments on the affected area, potentially improving patient outcomes. Additionally, all MRONJ sites were completely or mostly healed in this systematic review, further supporting the localized nature of the condition.

This study highlights several important considerations regarding the existing literature on dental implant survival affected by antiresorptive drugs. The majority of studies included in this review were case series and retrospective in nature, which may introduce limitations in establishing causality and controlling for confounding factors. Therefore, it is crucial to interpret the results with caution. To obtain more conclusive evidence, future prospective studies with appropriate control groups should be conducted to accurately assess the impact of antiresorptive drugs on implant outcomes. Some of the included studies had small sample sizes, which may limit the statistical power and potentially lead to a failure to detect significant results. Additionally, potential reporting bias should be taken into account, as success cases may not always be reported, potentially underestimating the true success rate of implants in patients using antiresorptive drugs. It is recommended to conduct more randomized controlled trials in the future to minimize bias. Lastly, the follow-up periods in the included studies may not have been sufficiently long to accurately reflect the rate of medication-related osteonecrosis of the jaws. Since MRONJ can develop over an extended period, longer follow-up durations are necessary to accurately capture its occurrence.

## 5. Conclusions

This systematic review highlights the significant impact of antiresorptive drugs on osseointegrated implants, with MRONJ identified as a leading cause of implant failure. Peri-implantitis was found to be a potential trigger for MRONJ. Regular monitoring and maintaining good periodontal health, particularly within the first three years of antiresorptive drug therapy initiation, are crucial for implant success. Treatment approaches for MRONJ include explantation, sequestrectomy, antibiotics, and chlorhexidine, with a high likelihood of complete healing observed at necrotic sites. These findings underscore the importance of careful assessment, monitoring, and appropriate management strategies for patients who plan to undergo antiresorptive drug therapy, as well as those with a family history of osteoporosis, to optimize dental implant outcomes. It is imperative that patients prescribed with antiresorptive drugs are equipped with comprehensive information regarding the risks associated with MRONJ, particularly its localized impact around osseointegrated implant sites. A longer term of follow-up is recommended to identify and manage MRONJ around dental implants in an early manner. The understanding of this risk may form part of the informed consent procedure for patients with dental implants considering to receive antiresorptive treatment. By providing patients with this knowledge and implementing appropriate monitoring and preventive measures, healthcare providers can optimize patient safety and long-term success in implant dentistry.

## Figures and Tables

**Table 1:** PICO of the systematic review.

P	I	C	O
Patients	Intervention	Control	Outcome
Patients with dental implants	Initiation of antiresorptive drug therapy after osseointegration of implant	Patients who did not receive antiresorptive drugs	Incidence of medication-related osteonecrosis of the jaw (MRONJ)

**Table 2:** Summary of articles that entered the final review.

Author and Year	Study Type	Patients	Dental Implants	Primary Disease in ARD Patients (*n*)	Mean Time between Initiation of ARD and MRONJ
Pichardo et al. (2020) [13]	case series	14	34 implants	Did not specify	Median 24 months (range 7–20)
Holzinger et al. (2014) [14]	case series	3	8 implants	Did not specify	18 months (range 14–23)
Goss et al. (2010) [15]	case series	4	12 implants	All osteoporosis	34 months (range 3–61)
Massaad et al. (2022) [16]	case series	6	11 implants	4 osteoporosis 2 cancer	51 months (range 24–192)
Kwon et al. (2014) [17]	case series	3	5 implants	All osteoporosis	19 months (range 13–27)
Pogrel et al. (2018) [18]	case series	11	11 implants	Did not specify	58 months (range 24–156)
Shabestari et al. (2010) [19]	case series	14	20 implants	All osteoporosis	No MRONJ
Kim et al. (2020) [20]	retrospective cohort	80	344 implants	Did not specify	11 patients—≤12 months 10 patients—13–35 months 13 patients—≥36 months

**Table 3:** Risk of bias assessment +—Low risk of bias, ?—Unclear risk of bias, -—High risk of bias.

	Study Design		Patient Selection		Data Collection Method		Outcome Assessment		Statistical Analysis	
	Presence of Control Group	Blinding	Sample Size	Representative Sample	Standardized Data Collection	Complete Data Reporting	Objective Outcome Measures	Consistent Outcome Assessment	Appropriate Statistical Tests	Complete Reporting of Results
Pichardo 2020 [13]	+	-	+	?	+	+	+	+	-	?
Holzinger 2014 [14]	+	-	-	?	+	?	+	+	?	?
Goss 2010 [15]	-	-	+	+	?	+	+	?	-	+
Massaad 2022 [16]	-	-	-	?	+	?	+	+	-	+
Kwon 2014 [17]	+	-	-	?	+	+	+	+	-	+
Pogrel 2018 [18]	-	-	-	?	+	?	+	+	-	?
Shabestari 2010 [19]	+	-	-	?	+	+	+	+	-	?
Kim 2020 [20]	+	-	+	+	+	+	+	+	+	+

## Data Availability

The data presented in this study are available on request from the corresponding author.
